# Opioid antagonist treatment in alcohol use disorder: extinction, reward memory, and the possible importance of timing

**DOI:** 10.3389/fnins.2026.1898127

**Published:** 2026-07-31

**Authors:** Iiro P. Jääskeläinen

**Affiliations:** Brain and Mind Laboratory, Department of Neuroscience and Biomedical Engineering, Aalto University School of Science, Espoo, Finland

**Keywords:** alcohol use disorder, extinction learning, memory reconsolidation, nalmefene, naltrexone, opioid antagonists, reward memory, timing-dependent effects

## Abstract

Opioid receptor antagonists, particularly naltrexone and nalmefene, are among the best-supported pharmacotherapies for alcohol use disorder (AUD), yet their clinical efficacy remains modest and highly heterogeneous. The mechanisms through which transient opioid receptor blockade produces sustained therapeutic benefit remain incompletely understood. Classical explanations emphasize attenuation of alcohol reward and suppression of craving, whereas extinction-based models propose that therapeutic effects emerge when alcohol consumption occurs under opioid receptor blockade, leading to weakening of conditioned drinking behavior. Clinical findings from targeted or “as-needed” treatment paradigms are broadly consistent with this interpretation. However, available observations are not fully explained by extinction alone. Preclinical animal studies suggest that subsequent alcohol drinking may depend critically on the timing of antagonist administration relative to alcohol exposure, including effects observed when opioid antagonists are administered immediately after drinking. Such findings raise the possibility that post-reward memory processes, including interference with reward-memory consolidation or reconsolidation, may contribute to therapeutic effects. Alternative explanations, including conditioned taste aversion, must also be considered, although the broader human literature more strongly supports modulation of reward and craving than generalized aversion. This mini-review integrates preclinical, clinical, and contemporary memory-neuroscience perspectives on opioid antagonist treatment in AUD. Improved understanding of these timing-dependent mechanisms, and their potential translational relevance to humans, may help explain clinical heterogeneity and guide the development of more targeted pharmacotherapeutic strategies.

## Introduction

Alcohol use disorder (AUD) is among the most prevalent and burdensome neuropsychiatric disorders worldwide, contributing substantially to premature mortality, disability, medical comorbidity, psychiatric illness, and social harm (World Health Organization, 2018; Rehm et al., 2009). Alcohol use has been estimated to account for 5% of the global burden of disease and injury; it also imposes major economic costs through healthcare utilization, lost productivity, accidents, and alcohol-related social harms (Rehm et al., 2009). In Europe, alcohol-attributable societal costs are estimated to exceed €100 billion annually, depending on methodology and included cost categories (Anderson and Baumberg, 2006).

Although psychosocial interventions remain central to treatment, recent meta-analytic evidence indicates only modest improvements in abstinence outcomes and no reductions in drinking frequency or quantity (Ghosh et al., 2024). Accordingly, search for pharmacological approaches has become increasingly important, particularly for relapse prevention and reduction of heavy drinking (Kranzler and Soyka, 2018). Among approved pharmacotherapies, opioid antagonists occupy a distinctive position. Unlike aversive agents such as disulfiram or glutamatergic approaches such as acamprosate, opioid antagonists target neural reward systems implicated in alcohol reinforcement (Koob and Volkow, 2010). Ethanol has been shown to stimulate the release of endogenous opioid peptides, including *β*-endorphin, thereby indirectly engaging the endogenous opioid system (Herz, 1997; Gianoulakis, 2009). Although this represents only one component of ethanol’s pharmacological actions (Koob and Volkow, 2010), it provides a compelling mechanistic rationale for the therapeutic efficacy of opioid receptor antagonists.

### Clinical efficacy and response heterogeneity

Clinical evidence indicates that opioid antagonist treatment for AUD produces reproducible but heterogeneous therapeutic effects. Early randomized trials yielded mixed findings, with some studies reporting reduced relapse or heavy drinking (Volpicelli et al., 1992; O’Malley et al., 1992), whereas others found weaker or even negative outcomes (Chick et al., 2000; Krystal et al., 2001). Subsequent meta-analyses and reviews have generally confirmed statistically significant but clinically modest average efficacy, particularly for reducing return to heavy drinking rather than consistently promoting complete abstinence (Rosner et al., 2010; Maisel et al., 2013; McPheeters et al., 2023). The most recent one, encompassing 118 clinical trials and more than 20,000 participants, identified oral naltrexone as one of the best-supported pharmacotherapies for AUD, while also underscoring that effect sizes remain modest and individual treatment response highly variable (McPheeters et al., 2023). Taken together, the clinical literature in AUD supports genuine therapeutic efficacy in humans, but also indicates substantial variability in treatment response.

The inter-individual variability in opioid antagonist response may also reflect biological heterogeneity. Pharmacogenetic studies have linked the OPRM1 A118G polymorphism to therapeutic response to naltrexone (Oslin et al., 2003; see also Chamorro et al., 2012) and increased hedonic response to alcohol (Ray et al., 2012), consistent with the hypothesis that inter-individual differences in opioid antagonist efficacy may partly reflect differences in endogenous opioid signaling, though no robust pharmacogenetic predictor has entered routine clinical practice. These clinical observations further support the possibility that opioid antagonist treatment effects may arise through multiple partially overlapping mechanisms.

Unlike traditional clinical paradigms in which opioid antagonists were administered as continuous daily treatment while abstinence was encouraged (Volpicelli et al., 1992; O’Malley et al., 1992; Anton et al., 2006), an alternative therapeutic approach has emerged in which medication is administered in temporal association with anticipated drinking episodes or high-risk situations. This event-contingent strategy explicitly links opioid receptor blockade to situations in which alcohol consumption may occur rather than maintaining continuous receptor blockade. In a randomized controlled trial, targeted naltrexone treatment without prior detoxification was associated with reductions in relapse to heavy drinking, suggesting that temporally targeted antagonist exposure may be therapeutically relevant (Heinälä et al., 2001). In a subsequent study, non-treatment-seeking problem drinkers took targeted naltrexone in anticipation of high-risk drinking situations or craving episodes, resulting in reduced drinking intensity (Kranzler et al., 2009). Additionally, a randomized placebo-controlled trial in individuals with mild to moderate AUD demonstrated that targeted oral naltrexone, administered in anticipation of drinking occasions rather than as continuous daily treatment, reduced binge drinking and alcohol craving (Santos et al., 2022).

A related but pharmacologically distinct opioid antagonist, nalmefene, has also been evaluated clinically in alcohol dependence in targeted “as-needed” treatment paradigms. Randomized studies reported reductions in heavy drinking and total alcohol consumption when nalmefene was taken in anticipation of drinking risk (Mann et al., 2013; Gual et al., 2013; van den Brink et al., 2014), although subsequent meta-analyses questioned the magnitude and robustness of these benefits (Palpacuer et al., 2015). While these studies do not establish a specific learning-based mechanism, they independently support the broader clinical relevance of temporally linked opioid antagonist administration.

Human laboratory studies likewise demonstrate reductions in subjective alcohol reward, cue reactivity, and craving under opioid antagonist treatment (Swift et al., 1994; Monti et al., 2001; Ray et al., 2012). More broadly, opioid receptor blockade has been shown to attenuate emotional responses to both positively and negatively valenced stimuli in humans, suggesting that endogenous opioid signaling contributes to affective processing beyond alcohol-specific reward (Heiskanen et al., 2019). In summary, evidence for targeted opioid antagonist administration spans populations ranging from alcohol-dependent patients without prior detoxification (Heinälä et al., 2001) to non-treatment-seeking problem drinkers (Kranzler et al., 2009) and individuals with mild to moderate AUD (Santos et al., 2022). However, whether such approaches are equally appropriate across the full spectrum of AUD severity remains an open question.

### Preclinical basis for the interventions

Early preclinical work in animal models implicated endogenous opioid mechanisms in alcohol reinforcement across multiple species. A landmark early study demonstrated that naltrexone reduced voluntary intravenous ethanol self-administration in rhesus monkeys in a dose-dependent manner, thus providing the first direct evidence that opioid receptor blockade could diminish alcohol’s reinforcing effects (Altshuler et al., 1980). Subsequent studies have extended these observations in both rodents and primates. In an operant rat self-administration model designed to distinguish alcohol-seeking (“appetitive”) behavior from alcohol consumption itself (“consummatory” behavior), Sharpe and Samson (2001) found that naloxone reduced ethanol intake primarily by shortening ongoing drinking, suggesting that opioid-receptor antagonism may alter the reinforcing consequences of alcohol consumption. Parallel findings emerged in non-human primates. Williams et al. (1998) demonstrated that naltrexone reduced ethanol self-administration in rhesus monkeys, further strengthening the evidence that endogenous opioid signaling contributes to alcohol reinforcement in species phylogenetically closer to humans. Shelton and Grant (2001) subsequently examined naltrexone in a multiple-schedule cynomolgus monkey self-administration paradigm in which the animals could self-administer ethanol during designated sessions, confirming suppression of ethanol-maintained responding under opioid receptor blockade. Together, these studies established robust preclinical evidence that opioid antagonists can acutely reduce alcohol-reinforced behavior across diverse animal paradigms and species. However, the mechanisms through which these observations relate to therapeutic effects in humans remain incompletely understood.

### Extinction of learned behavior as a mechanistic explanation

Some preclinical animal studies have proposed extinction of learned behavior as one potential mechanistic explanation for the therapeutic effects of opioid antagonists in AUD (Sinclair, 1990, 2001). According to this interpretation, therapeutic benefit may arise not merely from acute suppression of alcohol reward, but rather from extinction of alcohol-related conditioned behavior when alcohol consumption occurs under opioid receptor blockade. Specifically, in this theoretical framework, alcohol is consumed, expected reinforcement is attenuated relative to expectation, and thus conditioned drinking behavior gradually weakens (Sinclair, 1990, 2001). Notably, what is distinctive in this line of work was not the general observation that opioid antagonists can suppress alcohol consumption, but the explicit mechanistic emphasis on learning processes and the temporal relationship between receptor blockade and alcohol exposure. In this theoretical framework, the critical question shifted from whether opioid antagonists reduce drinking to under what behavioral conditions enduring reductions in alcohol-seeking and drinking might emerge.

The extinction of drinking behavior model is conceptually consistent with the “as-needed” opioid antagonist treatment paradigms described above, in which medication is taken before anticipated drinking rather than continuously under abstinence-oriented conditions (Heinälä et al., 2001; Kranzler et al., 2009; Santos et al., 2022). It may also help explain why some early daily-dosing clinical trials yielded inconsistent or negative outcomes (Chick et al., 2000; Krystal et al., 2001), since extinction-based learning would require alcohol-drinking behavior to occur under receptor blockade, whereas antagonist treatment administered primarily during abstinence would provide limited opportunity for such extinction of learned drinking behaviors. Importantly, while these clinical observations are conceptually consistent with an extinction-based account, they do not directly establish extinction learning as the mechanism underlying therapeutic efficacy in humans.

Notably, even in abstinence-oriented daily dosing trials, therapeutic effects may have arisen partly through relapse drinking occurring under opioid receptor blockade, thereby creating opportunities for learning-dependent mechanisms (Volpicelli et al., 1992; O’Malley et al., 1992). In these early studies, patients were encouraged toward abstinence, yet relapse drinking was not uncommon, and the observed benefits were expressed primarily as reduced relapse severity, delayed return to heavy drinking, or lower drinking intensity rather than universal maintenance of abstinence (Volpicelli et al., 1992; O’Malley et al., 1992). Conversely, later daily-dosing studies with weaker or negative outcomes may have provided fewer effective opportunities for such learning, whether due to poor adherence, differences in treatment context, or other clinical factors (Chick et al., 2000; Krystal et al., 2001). Because these studies did not explicitly manipulate the temporal relationship between alcohol consumption and receptor blockade, the relative contribution of acute pharmacological effects (e.g., craving suppression or reward attenuation) versus learning-mediated mechanisms remains uncertain.

### Timing dependence and learning-based mechanisms

**Figure 1:**
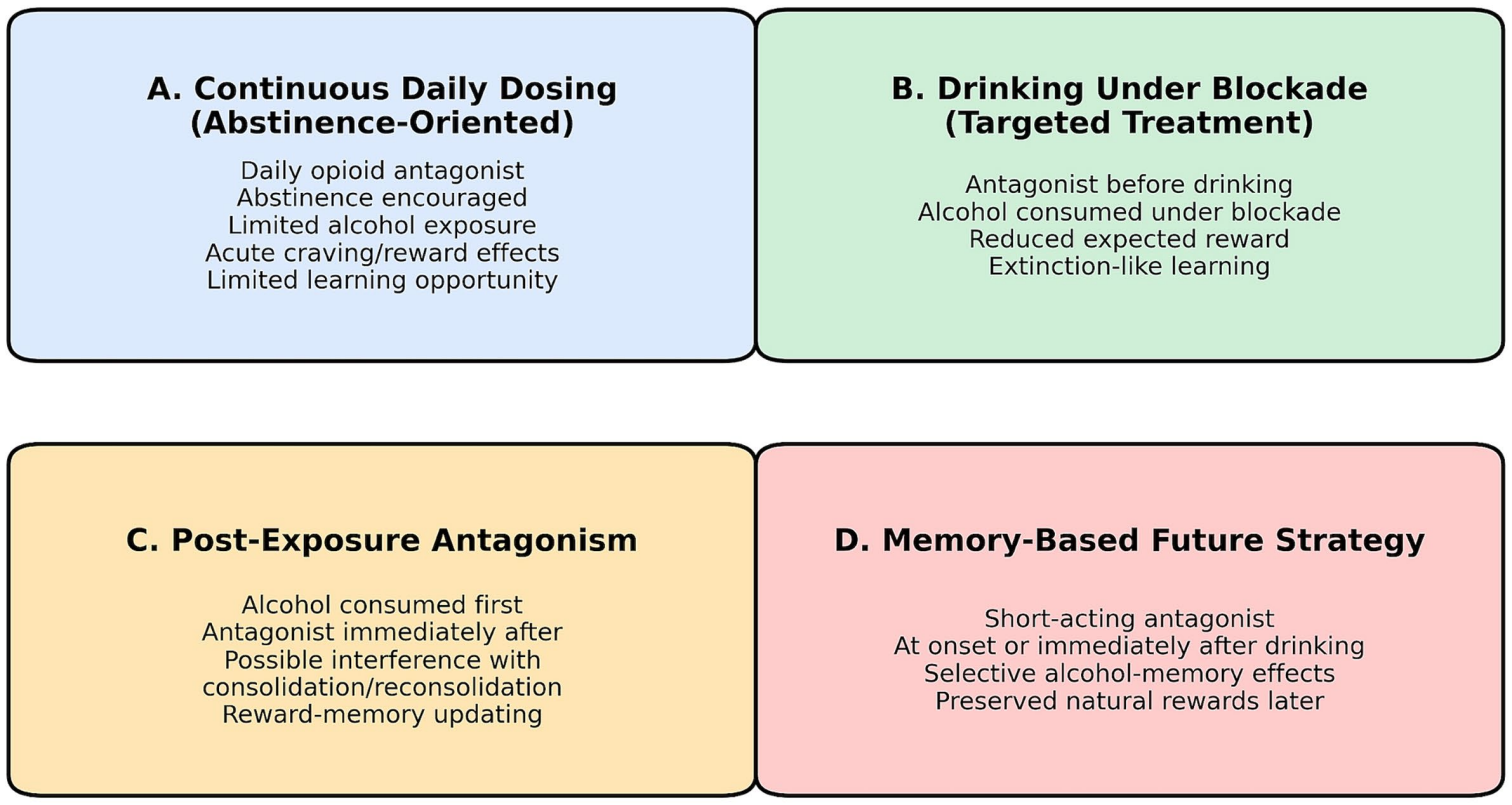
Four conceptual frameworks discussed in this review are illustrated. **(A)** Continuous daily opioid antagonist treatment administered during abstinence may primarily act through acute attenuation of alcohol reward and craving, while providing limited opportunity for learning-dependent modification of drinking behavior if alcohol exposure does not occur under opioid-receptor blockade. **(B)** Targeted administration before anticipated drinking allows alcohol consumption to occur under opioid receptor blockade and is consistent with extinction-based models in which attenuated reinforcement contributes to weakening of conditioned drinking behavior. **(C)** Antagonist administration immediately after alcohol exposure raises the possibility that reward-memory consolidation or reconsolidation processes may be modified. **(D)** A hypothetical future strategy based on short-acting opioid antagonists is presented as a conceptual example of temporally selective interference with alcohol-related reinforcement while minimizing broader effects on endogenous reward processing. Panels **(B–D)** represent mechanistic hypotheses that are not mutually exclusive.

Furthermore, early preclinical observations suggested that the behavioral effects of opioid antagonism on alcohol drinking depended critically on timing relative to alcohol exposure. The principal mechanistic frameworks discussed in this review are summarized in Figure 1. In rodent alcohol-drinking paradigms, naloxone administration well in advance of alcohol access prevented drinking under active receptor blockade and failed to produce sustained reductions in subsequent alcohol consumption; drinking levels remained comparable to baseline following treatment (Jääskeläinen, 1993; Sinclair and Jääskeläinen, 1995). By contrast, when naloxone was administered just prior to alcohol consumption so that it occurred under opioid receptor blockade, subsequent drinking behavior was reduced (Sinclair and Jääskeläinen, 1995), consistent with earlier reports supporting extinction-based interpretations of opioid antagonist effects on alcohol reinforcement (Sinclair, 1990).

A more conceptually provocative observation was that antagonist administration immediately after alcohol consumption also appeared to reduce subsequent drinking behavior (Sinclair and Jääskeläinen, 1995). This finding is less readily explained by classical extinction models requiring behavioral exposure under receptor blockade, and instead raises the possibility that post-exposure opioid antagonism may influence reward-memory consolidation or reconsolidation processes. While antagonism of opioid receptors during alcohol consumption could attenuate hedonic or reinforcing effects, producing classical extinction of conditioned behavior, antagonist exposure immediately after drinking could also interfere with post-reward memory consolidation processes required for maintaining reinforcement value (Nader et al., 2000; Lee et al., 2017). Whether this timing-dependent effect contributes to the therapeutic efficacy of opioid antagonists in humans remains unknown.

It is also possible that such findings may have been due to reactivation of established reward memories that become susceptible to pharmacological modification during reconsolidation (Huang et al., 2025; for non-pharmacological manipulations, see Xue et al., 2012). Memory reconsolidation refers to the phenomenon whereby previously consolidated memories, once reactivated, become transiently labile and susceptible to modification before being re-stabilized (Nader et al., 2000; Lee et al., 2017). In addiction neuroscience, retrieval and subsequent disruption of drug-associated memories have been shown to reduce later cue reactivity, craving, and drug-seeking behavior in experimental models (Milton et al., 2008; Huang et al., 2025; Xue et al., 2012). Applied to AUD, alcohol consumption itself could plausibly reactivate established reward-related associative memories. If alcohol-related reward memories are indeed rendered labile by alcohol re-exposure, opioid antagonism during this window could theoretically influence their subsequent reconsolidation. This interpretation remains speculative in the context of opioid antagonist treatment for AUD, yet it offers a possible mechanism that may help explain timing-dependent behavioral effects not readily accounted for by extinction alone.

Interestingly, disruption of drug-associated memory reconsolidation by NMDA receptor antagonism appears highly timing-dependent, with delayed administration failing to produce comparable effects (Milton et al., 2008). By contrast, early observations with opioid antagonists suggested that administration immediately after alcohol exposure could still influence subsequent drinking behavior (Sinclair and Jääskeläinen, 1995) and reconsolidation research has shown that previously consolidated memories are susceptible to pharmacological modification after retrieval (Nader et al., 2000). Whether these observations reflect a common memory-updating mechanism remains unknown and warrants further investigation.

### Alternative mechanisms of action

An alternative interpretation of the apparent post-exposure effects of opioid antagonism is conditioned taste aversion (CTA; Garcia et al., 1966). In the present context, CTA is discussed primarily as a broader framework for aversive learning rather than as a direct explanation of human alcohol consumption. From this perspective, reduced alcohol consumption following antagonist administration after drinking could reflect learned aversion to alcohol-associated post-ingestive effects rather than extinction of alcohol-seeking behavior or modification of reward-memory processes. Such an interpretation would be conceptually consistent with the classical CTA literature, in which delayed aversive consequences can become associated with ingested substances and subsequently suppress consumption (Garcia et al., 1966; Bernstein, 1999). This possibility deserves consideration, particularly in interpreting early rodent findings involving post-consumption antagonist administration. Supporting the plausibility of aversive mechanisms, human experimental work has shown that naltrexone can increase nausea, including during alcohol exposure, although such effects are not specific to alcohol-antagonist interaction alone (Jääskeläinen et al., 1998). However, the broader human literature does not strongly support a generalized aversive mechanism as the primary explanation for opioid antagonist efficacy in AUD. Human laboratory studies more commonly report reductions in alcohol craving and subjective reward rather than strong induction of aversive alcohol experiences (Swift et al., 1994; Monti et al., 2001). Thus, while aversive associative learning cannot be excluded as a contributing factor in some preclinical paradigms, it appears unlikely to provide a sufficient general explanation for the clinical effects of opioid antagonist treatment.

### Potential future directions

Interactions between opioid antagonist pharmacotherapy and psychosocial treatment may offer additional mechanistic clues. In an early randomized trial, O’Malley and colleagues observed that naltrexone efficacy varied according to psychotherapy context: overall abstinence outcomes were strongest with supportive therapy, whereas among patients who initiated drinking, the combination of naltrexone and coping skills therapy was associated with lower relapse risk (O’Malley et al., 1992). In a subsequent study, both naltrexone and cognitive behavioral therapy improved outcomes, but combining cognitive behavioral therapy with naltrexone did not produce dramatic additive benefits (Anton et al., 2006). Yet another study has shown beneficial interactions between naltrexone and coping skills therapy (Heinälä et al., 2001). These findings suggest that psychosocial context may influence opioid antagonist efficacy, but the nature of this interaction remains an open question. If therapeutic effects in humans depend partly on learning processes occurring during alcohol exposure under receptor blockade, specific behavioral interventions could plausibly enhance—or alternatively constrain—such mechanisms.

Furthermore, if opioid antagonist efficacy in AUD depends partly on selective interference with alcohol-related reward-memory formation or updating, this raises an intriguing pharmacological question regarding optimal drug kinetics. Both naltrexone and nalmefene have relatively prolonged durations of action, resulting in sustained opioid receptor blockade extending well beyond the period of alcohol exposure. Such prolonged blockade may be advantageous if therapeutic efficacy primarily depends on suppression of craving or attenuation of alcohol reward across extended time windows. However, if a key mechanism involves disruption of reward-memory consolidation or reconsolidation specifically linked to alcohol exposure, a different pharmacokinetic profile might theoretically be preferable. In principle, a rapidly acting but short-duration opioid antagonist administered at the onset of drinking—or potentially immediately thereafter—could selectively interfere with alcohol-related reinforcement processes while minimizing broader suppression of endogenous opioid-mediated reward responses associated with other adaptive behaviors, such as social interaction, exercise, or food reward. At present, this possibility remains speculative, but it illustrates how improved mechanistic understanding could eventually inform more precisely targeted pharmacotherapeutic strategies.

## Discussion

Opioid antagonist treatment for AUD represents one of the more biologically plausible pharmacotherapeutic approaches currently available, yet its clinical effects remain modest on average and highly heterogeneous between individuals (Rosner et al., 2010; McPheeters et al., 2023). Over the past three decades, both preclinical and clinical studies have consistently supported the conclusion that opioid receptor blockade can reduce alcohol-reinforced behavior, alcohol craving, and heavy drinking outcomes (Altshuler et al., 1980; Swift et al., 1994; Heinälä et al., 2001). However, the mechanisms through which transient receptor blockade translates into sustained behavioral change remain incompletely understood.

Extinction-based interpretations offer an intuitively appealing and clinically relevant explanatory framework, particularly in light of targeted treatment paradigms in which opioid antagonists are administered in temporal association with anticipated drinking (Sinclair, 1990; Kranzler et al., 2009; Santos et al., 2022). Yet available observations may not be fully explained by extinction alone. Early timing-dependent preclinical findings, including suppression of later alcohol drinking following antagonist administration immediately after alcohol exposure (Sinclair and Jääskeläinen, 1995), raise the possibility that post-reward memory processes may also contribute. Contemporary neuroscience concepts such as memory consolidation and reconsolidation provide plausible mechanistic frameworks for such effects (McGaugh, 2004; Lee et al., 2017), although direct evidence in the specific context of AUD, especially in humans, remains limited.

The broader clinical literature likewise supports mechanistic pluralism rather than a single explanatory model. Therapeutic effects may plausibly reflect interacting contributions from acute craving suppression, attenuation of alcohol reward, extinction-like learning, and potentially reward-memory updating processes, with relative contributions varying across individuals and treatment contexts (Monti et al., 2001; Ray et al., 2012). This may help explain why clinical outcomes remain heterogeneous despite strong neurobiological rationale.

Future studies could examine the temporal relationship between alcohol exposure and opioid receptor blockade experimentally rather than as an incidental treatment feature. Controlled human laboratory paradigms and translational studies capable of distinguishing acute pharmacological effects from learning-dependent mechanisms might be particularly informative. A more mechanistically precise understanding of when and how opioid antagonists modify alcohol-related behavior in humans could ultimately improve both patient selection and treatment strategy in AUD.
